# “This Is Not the Hill to Die on. Even if We Literally Could Die on This Hill”: Examining Communication Ecologies of Uncertainty and Family Communication About COVID-19

**DOI:** 10.1177/0002764221992840

**Published:** 2021-06

**Authors:** Rachael A. Hernandez, Colleen Colaner

**Affiliations:** 1 University of Missouri, Columbia, MO, USA

**Keywords:** family communication, COVID-19, communication ecology

## Abstract

As information about the public health risks surrounding COVID-19 continues to shift over time, families communicate to navigate this ongoing uncertainty. For example, families must interpret inconsistent media and public health messages about COVID-19, which may in turn have implications for health risk behavior. Adding to this complexity, household structures and routines are adapting in response to COVID-19. Adult family members in some families may suddenly experience extreme physical proximity, while others must coordinate to make decisions about their health and prevention behaviors while maintaining physical distance. Furthermore, members of these families must balance relational maintenance while communicating to assess and avoid health risks. The ongoing ambiguity of information about COVID-19 means that these relational processes must be managed in the midst of chronic uncertainty. The current study uses semistructured interviews and interpretive analysis to understand how adult children (aged 23-51 years) manage chronic uncertainty about COVID-19 in communication with their parents. Findings explore themes of navigating information about COVID-19 risks and protections, managing uncertainty management about media and political messages, and accepting time-related uncertainties.

On January 20, 2020, the first recorded case of the novel coronavirus (COVID-19) in the United States was reported in Snohomish County, Washington (Holshue et al., 2020). Two months later in mid-March, local and state governments enacted laws and recommendations for social distancing to prevent the spread of the virus. These policies and recommendations ranged widely in stringency, from stay-at-home orders in New York City, to mask recommendations put in place by local businesses. Over the course of the COVID-19 pandemic, public health groups, media organizations, and governmental agencies have produced incongruous messages regarding the nature and prevention of COVID-19 (J. L. Guest et al., 2020). These inconsistent messages created widespread uncertainty about the virus.

Family communication has the potential to play an important role in how individuals move through and recover from a crisis (Houston et al., 2017; Saul & Landau, 2004). Importantly, families are embedded in larger societal structures, with family systems interacting with local, regional, and national organizations. COVID-19 has heightened the interdependence of families and surrounding ecologies in constructive and constraining ways. When framed positively, family communication plays a role in bolstering community resilience (Houston et al., 2017). Given the incoherent communication efforts of various levels of government regarding COVID-19 (J. L. Guest et al., 2020), family communication is likely of heightened importance during this crisis. However, new difficulties have emerged for families during this pandemic. Families now face various unprecedented challenges such as sudden cohabitation in confined spaces or the need to coordinate decisions about their health over extended physical distances. Families must navigate all of these challenges while managing the uncertainties created by changing or ambiguous information about COVID-19 from various sources.

The current study examines the interdependence of family systems and surrounding resilience-bolstering community entities with an eye toward uncertainty management. Situating family relationships within the communication ecology perspective (Spialek & Houston, 2019) points toward tensions between individual and group dynamics with regard to micro-, meso-, and macro-resources. Conflicting approaches between adult children and their parents with regard to using community resources can prompt uncertainties that must be managed through family communication. Thus, the current study integrates uncertainty management theory (UMT; Brashers, 2001) with the communication ecology perspective (Broad et al., 2013) to illuminate how parent–adult child dyads experience and manage uncertainty related to COVID-19. Below, we first introduce UMT particularly within the context of disasters. We then explain the communication ecology perspective (Broad et al., 2013) to explore how parents and their adult children utilize resources to cope with COVID-19 related uncertainty.

## Uncertainty During Disaster Events

We define uncertainty as the inability to predict or understand the meaning of certain aspects of an individual’s environment (Bradac, 2001). Communication can either stimulate uncertainty or be a result of uncertainty (T. Afifi & Afifi, 2015). During a disaster, experiences of uncertainty may be short-term and occur immediately following a crisis event, or alternatively, may be long-term, and considered to be chronic uncertainty (W. A. Afifi et al., 2012; Brashers, 2001). For example, in the case of the U.S. terrorist attacks on September 11, 2001, the unpredictability of the attack heightened the resulting trauma of the event because for many individuals these events were unfathomable and changed their worldview (Saul & Landau, 2004). Conversely, in the case of the COVID-19 pandemic, the loss has been ongoing over the course of months, and with no certain end. This ongoing, long-term uncertainty can be understood as chronic uncertainty (Brashers, 2001).

Communication research often aligns with an “ideology of uncertainty reduction,” assuming that people want to reduce their uncertainty, and predicting that more information is the remedy for uncertainty-related distress (T. Afifi & Afifi, 2015, p. 43). More specifically, in the context of a crisis, uncertainty is often perceived to be best managed by providing individuals with more information (W. A. Afifi, 2010). However, the provision of information, especially when that information is inconsistent, can also increase uncertainty. Furthermore, at times individuals may seek to increase uncertainty, particularly in a crisis, as a way to keep at bay the difficult feelings that can come with unpleasant news (T. Afifi & Afifi, 2015). Thus, an alternative lens through which to view experiences of uncertainty is the UMT, which focuses on flowing through uncertainty, rather than reducing it (Babrow & Matthias, 2015). Strategies of uncertainty management include seeking or avoiding information, adapting to chronic certainty, eliciting social support, and balancing uncertainty management with other tasks (Brashers, 2001). These uncertainty management strategies are particularly important when individuals must make important health-related decisions (Dean & Davidson, 2018). A useful framework through which to understand the numerous broad factors that influence disaster communication is the communication ecology perspective.

### The Communication Ecology Perspective

The communication ecology perspective considers various levels of communication systems and resources available to individuals to help them gain knowledge and reach their goals (Broad et al., 2013). For example, relevant communication ecologies may include micro-level systems and resources, such as communication among nearby residents or communication in families (Spialek et al., 2019). Meso-level resources include messages on the local news or outreach from a local relief organization. Macro-level resources include communication sources such as national media. In the context of a disaster, the ecological layers of various systems and resources are integral to understanding and fostering community resilience, or the ability to “bounce forward” (Houston et al., 2015, p. 270). The current study follows Houston’s (2018) suggestion to focus research efforts on understanding the content of “interpersonal disaster talk” (p. 20), specifically on the interplay between disaster talk in families communication and other levels of the COVID-19 communication ecologies.

During a crisis, individuals rely on information from public health groups, media organizations, and governmental agencies. These messages are often discussed and interpreted via interpersonal communication. Research suggests that interpersonal communication strengthens the effects of media messages about health risk (Morton & Duck, 2001). Interpersonal communication may also shape knowledge about a pandemic (Ho et al., 2013), perceptions of the risks associated with a pandemic (Lin & Lagoe, 2013), and behavioral intentions to engage in protective behaviors, such as wearing a mask or avoiding crowded places. (Ho et al., 2013). Furthermore, for many people, family members are an essential source of social support and resilience during a disaster. However, the pandemic has created new challenges for families who must live under the same roof, or who must manage the challenges of the pandemic from a distance.

The current study specifically focuses on adult children because of the emerging need for many adult children and their parents to communicate to coordinate new living arrangements and to navigate parents’ inherent vulnerability to COVID-19 due to older age. For example, as of June 2020, 1 in 10 individuals aged 18 to 29 years reported moving because of the pandemic (Cohn, 2020), many of them returning to their parents’ home (Holder, 2020). Both multigenerational living and intergenerational communication from a distance may create conflict, subverting social support and potentially creating more distress in an already stressful time. The importance of the adult-child/parent dyad is heightened due to the need to navigate an abundance of information about the pandemic and make decisions for health. Furthermore, there are generational differences in perceptions of COVID-19 risk as well as the extent to which individuals are affected by the pandemic (Perna, 2020). Thus, this study employs communication ecologies as a sensitizing device to explain how adult children and their parents navigate uncertainties related to COVID-19.

**Research Question 1:** How do adult children manage ecological layers of uncertainty in communication with their parent(s) about COVID-19?

## Method

Given the need for intergenerational communication prompted by COVID-19, this study employed semistructured interviews to understand the COVID-19-related communication between parents and their adult children in families. After achieving institutional review board approval, participants were recruited online via Facebook, reddit.com, Instagram, and snowball sampling. Participants were individuals who are either living with at least one parent in the past 30 days as a result of social distancing or have communicated with at least one parent in the past 30 days about COVID-19. Interview questions focused on frequency of communication, communication successes and challenges, changes in communication over time, and the development of family emergency plan. The protocol included questions such as, “How do you and your parent(s) talk about the ongoing, changing messages about COVID-19?” and “Have you made a family plan in case of emergency?” While all of the questions specifically probed parent–child communication, some of the participants’ responses also included discussions of surrogate parents, siblings, spouses, and in-laws.

Seventeen interviews were conducted via Zoom Video Communications (Zoom), resulting in 279 pages of single-spaced interview text. The first author assessed saturation by recording her perceptions of the extent to which new information was gleaned from the data. Given the specific topic and the quality of the data, this study surpasses a suggested threshold of 12 interview participants (G. Guest et al., 2006). The first author also monitored the decreasing number and frequency of notes taken during the interviews, another sign of saturation (Tracy, 2012). Finally, in last third of the interviews, the first author engaged in member reflections, allowing participants to give feedback regarding the study findings and emerging themes (Tracy, 2012).

Participants ranged in age from 21 to 51 years, with an average age of 33 years. Interviews were conducted with individuals living across the United States, and one participant living in New Dehli, India. Eleven participants self-identified as White, three identified as Latinx, one identified as Asian, one identified as unknown, and one identified as “other.” Special attention was paid to recruiting individuals from diverse family structures, and adoptive families (1), stepfamilies (4), single-parent families (2), intact families (11), and chosen families (3) are represented in the sample. Nine participants were single, two were dating or in a relationship, and six were married. The interviews ranged from 30 minutes to 1 hour, with an average time of 50 minutes.

The first author wrote memos throughout the interviewing and analysis process, noting major themes and proposing relationships between the themes. The interviews were transcribed using an online transcription service, and the researchers used the qualitative analysis software Dedoose to sort and organize the data. Participants were given pseudonyms to protect their identities.

The transcripts were reviewed for the presence of statements related to uncertainty. Once it was determined that themes related to uncertainty were woven throughout the interviews, one researcher conducted a line-by-line analysis of the interview transcripts. which resulted in 11 primary codes illustrating uncertainty management (Charmaz, 2006). After developing these primary codes, the authors discussed discrepancies which resulted in combined and new codes. This interactive process led to the three primary themes found in the results. Next, the researchers engaged in axial coding, developing secondary themes that explained the relationship between the primary themes (Charmaz, 2006). Finally, the authors engaged in an iterative approach to analysis, interpreting the relationship between primary and secondary themes through the lens of UMT and the ecological model (Tracy, 2012).

## Results

The interviews revealed the role of uncertainty in several domains of the COVID-19 communication ecology. As a result of the pandemic, day-to-day communication and long-term coordination of family life intersected with macro-level ecologies of public health communication, media messages, and politics. The major themes involve (a) navigating uncertainties about COVID-19 risks and protections, (b) managing uncertainty management about media and politics, and (c) accepting time-related uncertainties. In each of these domains, participants described flowing through four strategies of uncertainty management: seeking information, avoiding communication, accepting uncertainty, and managing uncertainty management.

### Navigating Information About Risks and Protections

Inconsistent and unclear information about the prevention, symptoms, and detection of COVID-19 created confusion about how to protect against COVID-19 transmission. Three subthemes emerged in this theme. First, inconsistent public health and media messages about prevention made it difficult for the participants to convince their parents to adopt certain protective behaviors. Second, ambiguous information about COVID-19 symptoms made it difficult for families to predict if and when they should seek health care or a COVID-19 test. Third, some participants faced uncertainty from family members about the validity of their experiences with illnesses believed to be the result of COVID-19.

#### Coping With Inconsistent Information About COVID-19 Prevention

Participants described significant uncertainty about how to prevent the transmission of COVID-19. Some of this confusion stemmed from the volume and inconsistency of public health messages, which made it difficult to persuade family members to adopt protective behaviors. Georgina, who was temporarily living with her parents, said,

It becomes difficult to stress caution, because things seem to have changed. At first, we were supposed to be worried about surfaces, and now we’re finding out you don’t really have to be as worried about surfaces. And the usual thinking [is] that kids are little Petri dishes . . . but maybe they aren’t a good conduit of the virus. It’s all very confusing. It becomes very difficult to make a strong argument [with your parents], especially when it’s such an unknown.

For Georgina, this uncertainty was particularly troubling because she felt she could not persuade her parents that there was a need for protective measures.

Allie experienced family conflict surrounding inconsistent media messages about COVID-19. Allie felt that media-communicated uncertainty about COVID-19 made it difficult for her to coordinate a visit with her parents and convince them that her precautions were reasonable. She said,

[My mom] was very upset that I won’t give [her] a hug unless I have already showered and I’m wearing a mask. She had a problem with the fact that it would indicate I would not be comfortable in our home. I was like, “No shit, Sherlock. Pandemic.” . . . I don’t [think] that they are proceeding from the assumption that they have it. I get the impression it’s very much like dealing with AIDS, because if you look at the messaging, it was the same media hype, the same unknown . . . the same level of uncertainty.

In comparing the media coverage of COVID-19 to coverage of AIDS, Allie pointed to the uncertain messages about the risks and prevention of the virus which made it difficult for her parents to accept specific protective behaviors.

Carmen, who was also temporarily living with her parents, had a similar experience trying to keep her grandmother safe from COVID-19 by encouraging her to isolate from others. Carmen described how much of the public health information about COVID-19 was communicated through media channels. However, Carmen believed that media outlets obscured important information about COVID-19, and as a result, she and her parents had to provide her grandmother with accurate information. She said,

The media in America has been very, very weird of not speaking the truth sometimes, so we’ve had to educate our grandparents about, “Don’t fucking go inside anywhere unless you have to. I know it’s miserable” . . . Staying in this birdcage is torture, but if she wants to celebrate her 95th birthday, that’s what we have to do right now . . . [But] today, we got a phone call that she walked into a store to pick up a rotisserie chicken just because she couldn’t take it anymore. We had a big family blowout.

In Carmen’s family, when there was disagreement about information about COVID-19 or when the child’s words did not prove persuasive, family conflict ensued.

#### Interpreting Ambiguous Information About Symptoms

Recognizing the symptoms of COVID-19 is an important first step in finding health care and preventing the spread of the virus. However, many participants had difficulty interpreting which symptoms were serious. Most participants described getting information about COVID-19 symptoms from national television news channels, although one participant had done extensive research “on the dark web.” When asked how she and her family were making decisions about getting health care from a distance, Ayla explained how she had sought information from reddit.com and a website that aggregated COVID-19 symptom data from around the world. She said,

We’ve been just doing a ton of research. But . . . even though everyone’s talking about fever, bowel symptoms have shown up in 30% of cases . . . in the beginning, it was like if you’re coughing and you can’t breathe that was the telltale sign, but there’s actually so many signs . . .

Ayla felt that the most accurate and reliable information about the symptoms of the virus came directly from data on the internet. However, the information Ayla learned from this website was characterized by the same inconsistency as information from national news sources.

Another participant, Luis, watched national news with his parents every night, and they followed the varying symptoms of COVID-19. Luis said,

My dad had CNN on at night, every single night, that’s specifically when they would do the COVID-19 updates. Thank God it was never Fox News. Every so often we would discuss what’s new in COVID-land. At one point we were discussing, it’s weird how there’s this disconnect between what symptoms are, it feels like it’s starting to spiral out of control . . .

Like many participants in the study, Luis and his family watched the news together, and discussed the ongoing, changing symptoms of COVID-19. Luis’s statement that the disparate symptoms of COVID-19 were starting to “spiral out of control” illustrates how seeming impossible it is to know whether certain symptoms indicate that one is infected with the COVID-19 virus.

#### Facing Familial Ambivalence About One’s COVID-19 Status

Several of the participants felt that they had already been sick from the COVID-19 virus. These participants described their efforts to track and map their symptoms onto mediated information about COVID-19, seeking health care, and finding solace in their personal belief that their illness was caused by COVID-19. However, vague information about COVID-19 created some skepticism among family members that the illness had actually been caused by the COVID-19 virus. The family members’ denial subverted opportunities for social support, and left the participants feeling dejected.

One participant, Bill, had constructed a “chosen family” of extended family members, his wife, and in-laws. Bill was estranged from his biological parents, but believed that they broadly deny the existence of COVID-19. He was frustrated by his parents’ denial. Furthermore, his wife’s skepticism regarding his own experience of COVID-19 left him feeling hurt. Bill said,

Because I had all the symptoms, including fever, because I have a large amount of what appears to be antibody markers, my doctor’s basically like, “95%, you had it.” And, in a new disease where we didn’t have the tools to test, I guess that’s good enough for me. But there was definitely a month and a half there where I was like, “Am I crazy? What’s going on? . . . ”I literally went from . . . being able to run three miles to not being able to walk down the stairs without being able to breathe. . . . And, it’s been so nuanced . . . there’s so much misinformation out there . . . I can’t be 100% sure but my doctor is 95% sure [that my illness is COVID-19], I’m just going to call it [COVID-19] and just go from there. But, is that going to bother me for the rest of my fucking life? You better believe it is. [My wife] and I actually just had this conversation, because [my wife]’s not as sure as I am, and from a partner standpoint, that’s bothered me, because I’m having very real effects.

For Bill, his parents’ denial of the existence of the virus and his wife’s skepticism about his experience added layers of difficulty to the already difficult experiences of recovering from a new virus.

Another participant, Jill, had a similar experience to Bill, wherein some of her family members doubted that she was suffering from the COVID-19 virus. She said,

I got really sick in March, and we’re almost positive that it was COVID-19. It was all of the symptoms, breathing issues. It would not go away . . . [but] I didn’t qualify for a test . . . [And] some of my other family members, [were] like, “Oh, it sounds like you’re stressed. It sounds like you’re having anxiety.” “No, I’m sick. I know I’m sick.” It was a bit frustrating.

Jill spoke about this experience with a pained expression and felt hurt that she had to convince family members that her illness was valid. Later, Jill described how her communication with her mother gave her some comfort and that her mother’s belief that her illness was COVID-19 made her feel validated. These statements illustrate how uncertainties resulting from inconsistent information about COVID-19 can create tensions in families when someone is experiencing an illness. For these participants, part of their uncertainty management strategies involved acknowledging their hurt and trying to accept the skepticism of family members, while standing strong in their beliefs about the cause of their illness.

### Managing Uncertainty Management About Media and Political Messages

In addition to navigating health behaviors related to COVID-19, participants described several strategies by which to manage the onslaught of media messages about COVID-19. The 24-hour news cycle and availability of news on social media created a communication ecology wherein information (albeit inconsistent and ever-changing information) from macro-level ecologies was omnipresent and gave rise to strategies of seeking and avoiding information.

#### Allowing News to Bleed Into Family Communication

Participants who were temporarily living with their parents talked about how the national news was constantly on the television in their home. For others, the daily news was consistently a part of their long-distance communication with their parents. One participant, Phil, had been unexpectedly living with his parents, wife, and children at the beginning of self-isolation, a living arrangement that lasted for several months. Phil said,

[My parents] have the TV on all day anyway . . . it was always on the news because there was always the governor’s press conference on and the Trump task force press conferences were on. It was always something COVID-related we were watching or talking about.

Phil described how omnipresent macro-level governmental communication from televised news sources generated micro-level family communication about COVID-19.

Another participant, Allie, texted and called frequently with her parents who lived several hours away. She said,

We talk every day at least twice a day [about COVID-19] . . . because my dad won’t *not* watch coverage, right? So, we usually talk about it every day, even though they’ve realized it’s a sort of hot button. I’m on one end, Mom’s on the other, and dad’s stuck in the middle.

Like Phil, Allie and her parents’ communication about COVID-19 often centered around the national news. Even though news coverage was a point of tension between Allie and her parents, they continued to discuss the daily updates.

One participant, Kelly, strategically avoided political commentary about COVID-19 by not watching White House press conferences. However, the news consistently trickled into her family communication. She said,

I do not want to invite Trump into our relationship, and it feels like he’s here too much . . . it is a political issue in our context and that’s how we talk about it too. My husband and my dad [are] on their phone constantly, reading most of it through Facebook . . . a lot of it is like, “Guess what Trump did today?” Or, “Guess what they’re saying about it now?” . . . We’ll say, “Well we hit 100,000 deaths today. . . . People are on their phone and they blurt it out. Even prior to all of this anything with the political stuff, I like to consume [the news] intentionally when I’m in a mood to consume it. I’ve always had an issue with people just reading it and just interrupting my world, putting the information in my face.

For many participants, a glut of information about politics and COVID-19 was readily available via television news and social media. This information seeped into daily family communication, and at times led to conflict. Although for many participants, media was an important source of information to share with family members, they also described trying to control the amount and types of media that they consumed. This tension created a dilemma wherein participants found themselves trying to manage the stress of watching and talking about the news with their parents.

#### Avoiding Communication About News and Politics to Prevent Family Conflict

One important strategy for managing media messages about COVID-19 was simply avoiding exposure to news media. This strategy was particularly important because the overexposure of news media had the potential to create tension between family members. Some of the participants who were temporarily living with parents described deliberately avoiding the room with news playing on the television. This need for avoidance added another layer of how media messages and constrained physical environments have the potential to create tensions in families in the time of COVID-19.

One participant, Tori, was very frustrated with her mother-in-law. Tori believed that her mother-in-law was overzealous in her media consumption and fears regarding COVID-19. Exasperated, Tori said,

[My mother-in-law] watches the news every day. I don’t watch the news or read the news. And I roll my eyes when I start talking about it. . . . At the beginning [me and my in-laws] obviously talked about it more . . . but then after things started to settle by April, I was like, “Okay, I can’t talk about this anymore” . . . [I am] the literal poster child for unsuccessful communication with parents, because I don’t agree with [my husband’s] mom’s opinions at all.

In Tori’s family, news of COVID-19 was a source of frustration and open conflict in communication with her in-laws. Eventually Tori left her in-laws home and did not return.

Some of the participants said that they avoided communication with their parents about COVID-19 because of political tensions. One participant, Georgina, gave up on convincing her father of the seriousness of COVID-19 due to their political differences. Georgina said, “Sadly, it has become super political where it’s just not worth it, like this is not the hill to die on. Even if we literally could die on this hill, it’s fine.” For Georgina, the political tensions that arose led her to avoid communication about COVID-19.

Communication was tense among parents and children who disagreed about politics, but also created avoidance among children and parents who shared political views. Adan said, “I avoid talking to either of [my parents] for the two hours after Trump’s daily update. . . . Because they’re both a little worked up. And I just don’t need that extra stress right now.” For one participant, avoidance was followed by occasional venting about political frustrations. Carmen said, “We don’t really talk about it much together. We yell about policy makers. We scream about Trump every night at dinner, but that’s it.”

The dissemination of information about COVID-19 is often tightly interwoven with political communication, creating a partisan divide in matters of public health. Frequent presidential press briefings may be frustrating for individuals who disagree with the policies of the current administration, while those individuals who align with the current administration may feel it’s important to resist protective behaviors. The statements of these participants illustrate how this partisan divide creates difficulties not only for seeking out information but also for family communication about COVID-19. In addition to navigating public health information about COVID-19 risks and managing messages from media and governmental agencies, participants also described time-related uncertainties regarding the short-term and long-term outlook of the pandemic.

### Accepting Time-Related Uncertainties

The uncertainties surrounding COVID-19 were linked to both the short-term and the long-term realities of the pandemic. First, some participants had structured day-to-day schedules, while their parents found themselves with long stretches of free time. This disparate daily routine led to incongruous experiences of time among family members. Second, an unclear long-term timeline created tension among the family members. Finally, a potential strategy for coping with the long-term, chronic uncertainty faced by families was the development of a family emergency plan.

#### Coordinating Incongruous Circles of Time

The structure of daily work (or lack thereof) played an important role in family communication about COVID-19. In some families, parents and adult children had very different routines, and as a result, different experiences of time. For example, some of the participants had task-filled days and packed scheduled while their retired parents, freshly under the same roof, were facing less daily structure. In other cases, some individuals in the household were essential workers whose work occurred outside of the household, while their family members stayed at home in a slower, more independent daily routine.

One participant, Amber, was unexpectedly living with her parents as a result of the pandemic. Amber said,

I still have a structure. Even though the environment in which I’m working has changed, I’m still working, I’m still expected to be online. . . . For my parents, it all went away. For them, they are really in retirement. They’re on multiple pool leagues . . . my dad loves to fish, and he loves to meet his friends for coffee and go on motorcycle rides and do all these things. With the social distancing, all of that went away. If anything, time for them has really just stood still.

Even though they were living inside the same built environment, Amber’s daily routine stayed somewhat stable, and at times she felt “there wasn’t enough time in the day.” Her retired parents, however, did not have the same structure, and in the absence of their usual hobbies, time dragged on. This incongruous experience of time was also extant between Leena, a participant who was an essential worker, and her mother, who was retired and lived several hours away. For this participant, difficulties coordinating video calls created some tension in her family.

Another participant, Vin, was unexpectedly living with his retired father, wife, and two children. Vin’s father was with the family for a brief visit when a stay-at-home order was implemented. Vin described how a prolonged visit left his father with little to do, and a different experience of time. When asked about his experience of time, Vin said,

For [my father], it was really difficult because I’m working, my wife is working, and since the lockdown started we’ve been working more, instead of working less. . . . [My Dad] was getting a little—I won’t say depressed—but he used to sit in the balcony and two times I saw him chatting . . . I thought he was speaking to somebody. I told him, “Dad you’re getting old, you’re getting senile or what? What were you doing?” [he replied] “Nothing, I was just reciting a song.”

For Vin’s father, the rest of the family’s day churned on without him and he was left to entertain himself.

The need to work remotely cast many adult children into an uncertain role, however, their daily schedule often remained the same, filled with Zoom meetings and online education. On the other hand, their parents often did not have the same routine, and without the same daily tasks, had redefine their role in the household. For the most part, participants accepted this time-related uncertainty. For example, one participant who was unemployed and living with his parents found himself with little to do day-to-day, and but was content with not having a routine. In his household, the daily tasks unfolded around him, while he settled into a more relaxed pace of life. In other families, some parents took on new responsibilities such as cooking or babysitting, which gave the other members of the household space to navigate their new busy schedules at home. In addition to these day-to-day uncertainties, long-term, chronic uncertainty was ever-present in the lives of these families.

#### Traversing an Unclear Long-Term Timeline

It is difficult to forecast how COVID-19 will affect society in the long-term, creating chronic uncertainty among family members. One participant, Allie, experienced some conflict with her parents about how they would spend time in person in the future. Tensions emerged particularly because Allie generally accepted the chronic uncertainty, while other family members struggled with it. Allie said,

My parents didn’t like those conditions [that I only visit them while social distancing]. [They say] “I want you to feel comfortable here. I want it to be normal.” They’re like, “Will it take you a year to be comfortable here?” I was like, “Maybe.” As I said, World War II, you had four years of this shit. “What could you do? Nothing. You do what you had to.”

Allie had emotionally prepared herself for years of social distancing and privation. However, for her parents, this bleak outlook on family togetherness was unacceptable. Another participant faced similar issues; her parents struggled with the unclear long-term timeline, while she felt more comfortable accepting the chronic uncertainty. Georgina said,

The thing that’s most difficult about this, obviously, the deaths and the illness are very difficult. But I think for people dealing with the quarantine aspect of this, it’s just not knowing when it ends. That is something that makes both of [my parents] very anxious. I’m Generation X, I can hang out at home all day long, it doesn’t bother me at all, but my parents are both self-employed by our nonprofit. So, for them, it is a really nerve-wracking thing. Even though they’re pretty much retired, it’s still like part of who they are, that is something to worry about.

Whether they are under the same roof or communicating over a distance, when some family members struggle more with long-term uncertainty than others, tensions may arise. These statements illustrate some of the intergenerational differences among family members as they try to comprehend how the future will unfold.

#### Developing an Emergency Plan

Participants were asked if they had developed a family emergency plan regarding COVID-19. A few families had developed emergency plans including advance directives and strategies for accessing care at local health care organization, however, most had not. The need to develop an emergency plan involving local health care resources was salient for both parents and adult children living together, as well as families coordinating from a distance. When asked if she and her family had developed an emergency plan, Leena said,

Definitely not . . . I don’t think we have any plans. I actually, my emergency contact on most of my forms is my aunt and not my mother, mostly for vicinity. My aunt is 30 minutes out, whereas my mother is an hour and a half. Should everything be really urgent. . . . But yeah, no, I don’t think we have any kind of a plan.

As she discussed the emergency plan, Leena realized that her current emergency plan did not include communication with her mother, which may have been problematic.

For Luis, who was living in a two-bedroom apartment with his parents and two siblings, an emergency plan was an absolute necessity. Luis’s parents are essential workers, and the danger of bringing the COVID-19 virus home sparked the development of an emergency plan. Luis said,

That’s been something we’ve briefly discussed, because that’s something I want to know when I first got here . . . If it gets severe, then we do know which hospital to go to that will definitely admit us. Then in terms of home care, if we do have to not isolate at the hospital, we have the room set up with disinfectant. It’s not hazmat suits, but they basically have their own makeshift hazmat suit if they want to bring it out. So, they also have thought about if we have to take food, water, supplies, whatever, into the room, and then afterwards disinfecting, once the infection is contained.

For adult children and parents trying to manage their health and relationship in the pandemic, the development of an emergency plan could be a source of stress, or, alternatively a way to cope with long-term, chronic uncertainty. One participant, Ayla, reflected on how she felt the need to scramble at the beginning of the quarantine. With family living in Canada, New York, and London, she felt that it was difficult to coordinate between herself, her siblings, and her parents. She said, “I feel like nobody had a plan . . . if this happens again in the fall, I will 100% have plan A, B, and C just so that it’s not like I’m paralyzed.”

## Discussion

These results illustrate that uncertainty is woven throughout the ecology of communication about COVID-19, particularly in families. Participants identified four key strategies for managing uncertainty about COVID-19: seeking information, avoidance, acceptance, and managing uncertainty management. The findings from this study add to the theoretical literature on uncertainty management and communication ecology in disasters and hold several practical implications for health communication professionals. These are discussed in turn below.

### Theoretical Implications

Findings in the current this study extend existing knowledge of the theory of uncertainty management and the communication ecology approach in three ways. First, the findings of this study illustrate a variety of techniques to cope with uncertainty related to COVID-19. While information-seeking was discussed in some of the interviews, it was not the dominant uncertainty management strategy, contrary to other health-related uncertainties (Brashers et al., 2000; Dean & Davidson, 2018). T. Afifi and Afifi (2015) argue that it is important to focus on the role of relationships in uncertainty in scenarios where individuals must manage complex layers of information, rather than focusing strictly on the role of information. Our results demonstrate how uncertainty management strategies such as avoidance of political discussions and news helped adult children cope with uncertainties related to COVID-19 while simultaneously maintaining family relationships. Furthermore, chronic uncertainty about the risks and prevention of COVID-19 was woven together with family conflict in everyday interpersonal communication. For example, ambiguity about protective measures, the political issues surrounding COVID-19, and the ambivalence about the COVID-19 status of a family member created dilemmas and tensions in the family. These findings vivify the claims of uncertainty management theory that uncertainty can be both preferable and problematic.

Second, findings expand on the notion of uncertainty sources. Not all of the uncertainties in the current were informational. Some uncertainties involved the functions of everyday life at home. The chronic uncertainty of the long-term timeline of the pandemic brought some families together under one roof. The need for parents and adult children to suddenly live and work in the same space illustrates the axis of time in the convergence of the human environment and the built environment. Changes in the function of the home and disparate daily schedules created difficulties in ascertaining the meaning of their respective roles. The built environment, originally intended for either parents or their adult children, must now accommodate both. The human environment, which involves the acts of doing work (or not doing work) in the same built environment of the home, created incongruous experiences of time for parents and adult children. COVID-19 resulted in changes in the meaning and experience of home for adult children and their parents. These eccentric circles of time led to parents and children trying to meet each other’s needs with varying success.

Third, findings from the current study shed light on the interdependence of micro- and macro-level systems. To date, a significant body of research exploring communication ecologies in a disaster has emphasized the important role of micro- and meso- level structures in community resilience (e.g., individual storytellers and stories told by local media; Spialek & Houston, 2019). The current study further illustrates the interplay of micro- and macro- level systems and resources during a widespread crisis. Notably most of the participants did not reference community organizations when managing uncertainty about COVID-19, but rather emphasized larger-scale government and media organizations. This focus may be the result of the fact that the interviews took place at a relatively early stage of the pandemic. Over time, as COVID-19 cases grew in different localities, families may have shifted their attention to meso-level city health departments and local news. Yet this insight into early responses to the global pandemic showcases the multifaceted connections with the ecological model.

### Practical Implications

In addition to theoretical contributions, the current study also points to four important practical suggestions for families and practitioners. First, T. Afifi and Afifi (2015) highlight the fact that health communicators often assume that uncertainty is the result of too little information and thus rarely focus on helping individuals wade through and interpret a surplus of information. The current study shows how some individuals living through a pandemic cope with a surplus of information through avoidance, acceptance, and managing uncertainty management. These results show how an abundance of information is not always a virtue and may create conflict between adult children and their parents. Findings here suggest that health communicators should be mindful of information quantity when interacting with uncertain individuals, perhaps striving to focus on message clarity or information coping rather than sharing all available information.

Second, much of the extant research literature encourages parents to use media messages as an opportunity to broach difficult conversations with their children (Borra et al., 2003; Coyne et al., 2014). This study demonstrates that when media coverage of a disaster is omnipresent and overwhelming, these news stories have the potential to bleed into everyday family communication. This frequent communication about the news may displace communication between parents and adult children about their needs for emotional support. Individuals should be mindful of the relational impact of different preferences for the quantity or quality of media consumption. Political differences tend to generate barriers to relational solidarity, yet the negative effect of political differences on relationships can be mitigated through accommodative communication (Warner et al., in press). Accommodative communication involves topic selection, communicating respect about differing opinions, limiting self-disclosure on contentious topics, and expressing tolerance for others’ beliefs. Participants in the current study highlighted the political lightening rod that COVID-19 introduced into the family system. Thus, it is important for families to balance individual information needs with the information boundaries set by family members living in close proximity in order to protect relational solidarity.

Third, this study demonstrates the need for families to develop emergency plans to engage with meso-level community resources (e.g., health care organizations) in advance of a disaster. Advance directives and plans for managing a serious illness are already sensitive topics, and the need to broach these subjects during a pandemic intensifies the associated distress. Many of the participants in the current study found themselves without an emergency plan and felt that it was currently too stressful to initiate this conversation. However, even in times characterized by ongoing uncertainty, families may benefit by becoming attuned to moments where there’s less pressure or opportunities before a high-pressure event in which they can coordinate and make their emergency plans. For example, emergency planning may be appropriate before some family members face COVID-19 exposure at school or work, move to a new area, or face an anticipated change in medical coverage. This planning is particularly important for some groups. Black and Hispanic adults are less likely than White adults to have an advance directive (Huang et al., 2016; Portanova et al., 2017). Furthermore, research shows that in the absence of a plan, there are significant racial differences in life-sustaining and family-clinician communication (Muni et al., 2011; Wenger et al., 2001). The results clearly demonstrate that families would benefit from taking any opportunity to have these difficult conversations about their needs during a crisis and emergency plans. It may benefit families to have emergency plan “checkpoints” wherein several times a year, adult children and their parents develop or revise their emergency plan.

Finally, the interwoven links between politics, media, and interpersonal communication had the potential to create conflict between family members. Relationships can suffer in times of personal stress given the reduced capacity to engage in constructive conflict strategies (Rinaldi & Howe, 2003). As COVID-19 provides compounding stressors of work, education, and health risks, relationships may experience new tensions. Participants in the current study also experienced structural changes which offered new opportunities for conflict. For example, living with one’s parents in adulthood increases interdependence which in turns increases opportunities for conflict. Differing political and social values add to the conflict potential. Adult children experiencing tensions in their relationships with parents may benefit from professional counseling to process relational difficulty. Although family counselors tend to focus on romantic relationships or parents and dependent children (Sow & Friedman, 2020), the present study points to an expanded model of therapeutic care that can be supportive of diverse family structures.

## Limitations and Future Research

The COVID-19 pandemic and resulting social distancing efforts have created novel uncertainties for families who must communicate to asses COVID-19 risks and prevention. This study examines only a cross-section of individuals, and the interviews occurred in the first five months of the pandemic. Experiences of chronic, ongoing uncertainty will likely change as the months and years go on. Future explorations of uncertainty and communication ecology in disasters should examine how the uncertainty management in communication ecologies evolves over months and years. Finally, the racial makeup of the sample population for this study was not representative of the general population. Future research in this area should endeavor to represent individuals from many different minoritized groups.
